# Brain Damage and Repair: From Molecular Effects to Central Nervous System Disorders

**DOI:** 10.3390/biology10060489

**Published:** 2021-05-31

**Authors:** Olivier Armant, Christelle Adam-Guillermin

**Affiliations:** Laboratoire d’Ecotoxicologie des Radionucléides, Institut de Radioprotection et de Sûreté Nucléaire, IRSN/PSE-ENV/SRTE/LECO, Bâtiment 183, Cadarache, BP3, 13115 Saint Paul-lez-Durance, France; christelle.adam-guillermin@irsn.fr

Chronical exposures to biological, chemical and physical stressors can be particularly detrimental during the early phase of embryonic development, increasing the risk of brain dysfunctions after birth. Major advances have been made in our understanding of the mechanisms driving the development of a fully functional brain from a limited pool of stem cells, as well as how this organ can respond after injuries. However, further knowledge is required to understand how these processes are altered by environmental stressors, such as ionizing radiations, chemicals, stroke or viral infectious agents, and therefore be able to predict the etiology of neurological disorders. Ionizing radiation is a prototypical genotoxic stressor for which a deep scientific knowledge is available. This ranges from energy deposition of charged particles or photons on living tissues, to epidemiological studies on atomic bomb survivors in Hiroshima and Nagasaki. In this special issue, three original research articles highlight how novel, state-of-the art irradiation tools can be employed as alternatives to bulk whole body exposure, in order to delineate the consequences of cell depletion, on organ function. Namely, Takako Yusuda et al. took advantages of the external development of Medaka (*Oryzias latipes*) embryos and targeted irradiation with a collimated microbeam, to investigate how ablation of increasing numbers of embryonic cells at the blastula stage—a very early phase of embryonic development before gastrulation—can affect later developmental steps [1]. They demonstrated that loss of less than 10% of blastodermal cells induce a transient brain developmental delay, while a loss higher than 25% of blastoderm cells lead to embryonic lethality during the neurulation period. In another study, M. Suzuki et al. used the nematode in order to assess the dose-dependent effects of the central-nervous-system-targeted irradiation on the motility of *C. elegans* [2]. C. Serrano et al. targeted the neurogenic niche of stem cells located in the dentate gyrus of 10-day old postnatal mouse, and observed a non-linear dose response on long-term spatial memory in adulthood (three month after irradiation), using a spatial water maze test [3]. 

Knowledge on key biological processes like neurogenesis and cellular differentiation in both normal and pathological conditions is instrumental for predicting the occurrence of cognitive diseases from the early onset of key biological markers, as well as also to design novel drugs to avoid or delay detriments. In this issue, one review details the role of DNA damages in the occurrence of neurodegenerative disorders [4]. J.L. Yang et al. investigate how the well-known glucose uptake regulator glucagon-like peptide-1 (GLP-1), function as a key neurotrophic factor, promoting the neuronal differentiation of SH-SY5Y into physiologically mature glutamatergic and dopaminergic neurons [5]. In another paper that focuses on an animal model of Alzheimer’s disease, J. Mayordomo-Cava et al. show that the soluble form of Amyloid-β (Aβ) peptide Aβ_25–35_ alters the hippocampal network activity and results in memory deficit, when tested by the open field habituation test and the novel object recognition test [6]. Once formed, the brain can still be subjected to lesions, e.g., after an accident, leading to cerebral ischemia. In their paper, M. Tóth et al. review the mechanisms of acidosis-linked neuronal injury in cerebral ischemia and how nanomedicine can be used to both identify regions at risk and guide neuroprotective intervention after ischemic stroke [7].

Monitoring the exposures to toxicants and stressors throughout the entire lifespan, including the prenatal stage, as formalized in the concept of the exposome, highlights the methodological and conceptual challenges for considering the environment as a potential source of stressors that can impact heath. The huge diversity of chemical and physical agents produced by anthropogenic activities preclude the toxicological testing of every single stressor. A framework is thus needed to gather the existing and the forthcoming knowledge on the different stressors and their effects at different biological scales (molecular, cellular, individual), as well as to rationalize experimentations to decrease costs and construct predictive models for mixtures of stressors. The adverse outcome pathways (AOPs) is a formal framework that helps to assemble the current knowledge on well-accepted biological events that lead to disease progression. In addition, AOPs are proposed to build models that are useful for risk assessment. AOPs start with a molecular initiating event (MIE) defined as the first chemical or physical interaction of a stressor with its molecular target. Once initiated, the MIE leads to a series of downstream key events (KEs) at the molecular, cellular, tissue, organ, and individual level, which are necessary for the apparition of the adverse outcome (AO). However, building an AOP approach does not only involve making a compilation of the current scientific knowledge, it is also a collaborative exercise that requires the enrollment of recognized experts in their field to identify KE and build the weight of evidences between the initial MIE and the final AO. Such exercise is thus a good opportunity to share and normalize the knowledge, and also to bring consensus across a scientific community. The utility of such framework is exemplified in this special issue that covers how environmental stressors can perturb brain development and functions to translate into cognitive and behavioral phenotypes. Namely, M. Sachana et al. describe how the AOP approach can be used in the field of development of neurotoxicity of chemicals [8].
